# The association of serum immunoglobulins with cognition and dementia: the Rotterdam Study

**DOI:** 10.1007/s00415-022-11374-7

**Published:** 2022-09-19

**Authors:** Samer R. Khan, Amber Yaqub, M. Kamran Ikram, P. Martin van Hagen, Robin P. Peeters, Virgil A. S. H. Dalm, Layal Chaker, M. Arfan Ikram

**Affiliations:** 1 Department of Epidemiology, Erasmus University Medical Center Rotterdam, PO Box 2040, 3000 CA Rotterdam, The Netherlands; 2 Division of Allergy and Clinical Immunology, Department of Internal Medicine, Erasmus University Medical Center Rotterdam, Rotterdam, The Netherlands; 3 Department of Neurology, Erasmus University Medical Center Rotterdam, Rotterdam, The Netherlands; 4 Department of Immunology, Erasmus University Medical Center Rotterdam, Rotterdam, The Netherlands; 5 Division of Endocrinology, Department of Internal Medicine, Erasmus University Medical Center Rotterdam, Rotterdam, The Netherlands

**Keywords:** Dementia, Cognition, Immunoglobulin A, Immunoglobulin G, Immunoglobulin M, Epidemiology

## Abstract

**Background:**

Chronic inflammation is involved in the pathophysiology of dementia, but the association of serum immunoglobulins with dementia has been understudied and longitudinal data are currently lacking. We investigated the association of serum immunoglobulin (Ig) A, G, and M with cognition and dementia in a population-based cohort.

**Methods:**

This study was embedded in the Rotterdam Study. Participants with information on serum immunoglobulin levels, measured between 1997 and 2009, were followed for incident dementia until 2016. Assessment of cognitive function and dementia was performed according to validated tests and clinical criteria respectively. We studied the association between serum immunoglobulins with prevalent and incident dementia using logistic regression and Cox proportional hazards regression analyses respectively. We performed linear regression analyses to quantify the cross-sectional association of serum immunoglobulins with global cognition as well as separate cognitive tests. Analyses were adjusted for age, sex, lifestyle, and cardiovascular factors.

**Results:**

We included 8768 participants (median age of 62.2 years, 57% women, median follow-up 10.7 years). Overall, none of the immunoglobulins was associated with prevalent or incident dementia. Higher IgG levels were associated with lower scores of global cognition (adjusted standardized mean difference − 0.04; 95% confidence interval:− 0.06; − 0.02) and separate cognitive tests.

**Conclusion:**

In middle-aged and older individuals from the general population, serum Igs were not associated with prevalent or incident dementia, which may imply that serum Igs are not involved in the pathophysiology of dementia. Although higher IgG levels were associated with worse cognitive function, studies with longitudinal data should exclude reverse causation.

**Supplementary Information:**

The online version contains supplementary material available at 10.1007/s00415-022-11374-7.

## Introduction

Dementia is an umbrella term for a heterogeneous syndrome characterized by cognitive decline resulting in impairment of social functioning or activities of daily living [1]. Alzheimer’s disease (AD), the most prevalent dementia subtype, is characterized by cerebral deposition of the proteins Amyloid β (Aβ) and hyperphosphorylated tau, which induce gradual and permanent neuronal damage [2]. However, the pathogenesis of dementia is complex and not fully unraveled. Recent advances have contributed to the understanding that a chronic inflammatory state, involving a complex interplay between various components of the immune system, plays a key role in the development of dementia [3].

Pro-inflammatory cytokines including interleukins and tumor necrosis factor, and inflammatory proteins have been extensively studied in relation to AD [4, 5]. Pro-inflammatory cytokines have been reported to contribute to the pathogenesis and progression of dementia, possibly by promoting cerebral Aβ deposition, oxidative stress, and apoptosis, processes that can facilitate neuroinflammation [3, 6]. In contrast, the role of serum immunoglobulins (Igs) has been relatively understudied. IgA, IgG, and IgM are among the main classes of serum Igs in humans [7].

Several cross-sectional studies have investigated the relation between serum Igs and cognition and dementia [8–11]. The largest study to date (*n* = 122) reported higher IgA and IgG levels in cognitively impaired individuals compared to age- and sex-matched controls [9]. However, higher IgG and IgM levels were correlated with a better cognitive performance in dementia patients [8]. Studies on the relation between serum Igs and dementia reported inconclusive results as well [10, 11]. Nevertheless, randomized controlled trials (RCTs) embarked upon investigation of intravenous Ig (IVIG) supplementation as a treatment modality in AD. A meta-analysis of five RCTs was conducted to provide an overview of the safety and efficacy of IVIG in AD patients. Although safe, efficacy of IVIG in AD patients with regard to cognition could not be demonstrated, which has been speculated to be due to the advanced disease stage of the included patients or the relatively low employed IVIG dose [12]. However, it could also imply that there is no value of IVIG treatment in dementia.

To date, there are no longitudinal studies on the association between serum Igs and cognition and dementia, making it difficult to disentangle the temporality of the association. In addition, most previous studies were patient-based, had a small sample size, or did not consider potential confounders such as cardiovascular risk factors. Therefore, the association of serum Igs with cognitive function and dementia risk in individuals from the general population remains elusive. We aim to assess these associations in a large population-based cohort of middle-aged and older individuals. This may contribute to a better understanding of the pathophysiology of dementia and the rationale of IVIG treatment in dementia.

## Methods

### Study design and participants

This study is embedded in the Rotterdam Study, a large ongoing prospective population-based cohort study of middle-aged and older individuals residing in Ommoord, a suburb of the Dutch city of Rotterdam. Detailed information on the Rotterdam Study has previously been published [13]. In summary, the Rotterdam Study started in 1990 and had included 14,926 participants aged 45 and over by the end of 2008. The Rotterdam Study was extended with independent cohorts in 2000 and 2006. All participants are re-examined every 3–6 years. Re-examinations comprise home interviews and assessments at the research center in Ommoord. Additionally, continuous linkage with databases of general practitioners (GPs), hospitals, and regional pharmacies takes place. The Rotterdam Study has been approved by the Medical Ethics Committee of the Erasmus MC (registration number MEC 02.1015) and by the Dutch Ministry of Health, Welfare and Sport (Population Screening Act WBO, license number 1071272-159521-PG). For this study, we included participants of three independent cohorts of the Rotterdam Study (I-3, II-1, and III-1) that had measurements of serum IgA, IgG and/or IgM available in blood drawn between 1997 and 2009, had information on dementia, and had given written informed consent for follow-up.

### Assessment of serum immunoglobulins

Serum Ig levels were measured in venous blood drawn at the research center. The serum samples were subsequently stored at − 80 °C and thawed for assessment between 2016 and 2018. Serum IgA, IgG, and IgM were measured with an immunoturbidimetric assay (Tina-quant^®^ IgA/IgG/IgM Gen. 2, Roche Diagnostics GmbH, Mannheim, Germany). Coefficients of variation between batches (to assess time trends or batch effects) were 1.12–2.68% for each Ig. Coefficients of variation across batches (to assess assay precision) were 2.05–3.58% for each Ig. Reference ranges for adults according to the manufacturer’s protocol were 0.7–4.0 g/L for IgA, 7.0–16.0 g/L for IgG, and 0.4–2.3 g/L for IgM. Reference ranges based on the 2.5th–97.5th percentiles for our study population were 0.86–4.76 g/L for IgA, 6.20–15.10 g/L for IgG, and 0.28–2.64 g/L for IgM, as previously described [14].

### Assessment of cognition

Cognitive function was assessed at the research center through validated tests, as described previously [15]. Available cognitive tests were the Mini-Mental State Examination (MMSE), Stroop test, letter digit substitution test (LDST), word fluency test (WFT), Purdue pegboard test (PPB), and the word learning test (WLT). The MMSE is a well-known test of global cognitive function. The Stroop test consists of three subtests to evaluate the speed of reading, speed of color naming, and interference of automated processing and attention respectively. The LDST is a test for processing speed and executive function, whereas the WFT assesses the efficiency of long-term memory retrieval. The PPB assesses dexterity and fine motor skills and was performed with the right, left, and both hands and these scores were subsequently summed. The WLT consists of three tests for the assessment of verbal learning (immediate recall), retrieval from verbal memory (delayed recall), and recognition of verbal memory (recognition). A global cognitive score (G-factor) was obtained by performing a principal component analysis (PCA) of various cognitive tests, as previously described [15]. The variance of cognitive tests explained by first principal component, for which the term G-factor is used, was 47% in cohort III, 63.4% in cohort II, and 63.2% in cohort I. All cognitive test scores were available in the included cohorts, with the exception of PPB and WLT, which were only available in cohort III-1.

### Assessment of dementia

Assessment of dementia was performed at baseline and each consecutive follow-up visit. During the visit at the research center, participants were screened for dementia with the MMSE and the Geriatric Mental Schedule (GMS) organic level. Participants with a MMSE < 26 or a GMS > 0 underwent an examination and informant interview with the Cambridge Examination for Mental Disorders of the Elderly. Electronic linkage of the study database with medical records from GPs and the Regional Institute for Outpatient Mental Health Care provided additional continuous surveillance for dementia incidence for the entire cohort. A consensus panel led by a consultant neurologist, blinded for all other covariables including serum Igs, decided on the final dementia diagnosis in all cases in accordance with the DSM-III-R criteria for all-cause dementia and the NINCDS-ARDRA for the subtype of AD. Clinical neuroimaging was used as an aid to determine the subtype of dementia or to rule out other causes when needed. Dementia follow-up was completed until January 1, 2016.

### Assessment of other baseline covariables

Information on smoking status, alcohol consumption, and highest attained education was obtained through questionnaires during home interviews. Smoking status was categorized as never, former, or current. Alcohol consumption was measured in gram/day and categorized into none, mild (0–10 g/day), moderate (10–20 g/day), and heavy (> 20 g/day). Apolipoprotein ε4 (*APOE* ε4) carrier status was defined as the presence of one or two ε4 alleles. *APOE* ε4 genotyping was performed with a polymerase chain reaction (cohort I-3) or bi-allelic TaqMan assay (rs7412 and rs429358; cohorts II-1 and III-1) on coded DNA samples without knowledge on dementia status. Body mass index (BMI) was measured at the research center by dividing weight by height squared (kg/m^2^). Blood pressure was measured at the research center at the right arm and the average of two consecutive measurements was used. Hypertension was defined as a blood pressure of ≥ 140/90 mmHg or as the use of blood pressure lowering medication with the indication of hypertension. Diabetes mellitus (DM) was defined as a fasting blood glucose concentration of ≥ 7.0 mmol/L, a non-fasting blood glucose concentration of ≥ 11.1 mmol/L (in the absence of fasting samples), or as the use of pharmacological or dietary treatment for diabetes. Information on baseline stroke and coronary heart disease (CHD) was obtained through home interviews and medical records of GPs. CHD was defined as myocardial infarction, percutaneous coronary intervention, or coronary artery bypass grafting. Total serum cholesterol was measured in mmol/L with an automated enzymatic procedure. Use of medication that may influence serum Ig levels (systemic corticosteroids, antiepileptic drugs, angiotensin converting enzyme inhibitors, cytostatics, immunomodulators, and/or immunosuppressants) was retrieved during home interviews at baseline and coded according to the anatomical therapeutic chemical system.

### Statistical analyses

Serum Ig levels and cognitive test scores (except for G-factor and MMSE) were standardized to facilitate comparison of effect estimates (presented per standard deviation increase). Stroop test scores were furthermore inverted for interpretation, as lower scores reflect better cognition.

We assessed the association between serum Igs and prevalent dementia by binomial logistic regression analyses to obtain odds ratios (ORs) and 95% confidence intervals (95% CIs). Analyses were adjusted for potential confounders based on biological plausibility and previous comparable research [16, 17]. A first model was adjusted for age and sex. A second model additionally included smoking status, alcohol consumption, highest attained education, and *APOE* ε4 carrier status. A third model included covariables that may be mediators or confounders and comprised BMI, hypertension, total serum cholesterol, DM, history of CHD, and history of stroke additional to the first two models. There was no multicollinearity and the linearity of log odds assumption was met for all analyses. To limit the influence of transient outliers, we performed a sensitivity analysis by excluding participants with serum Ig levels outside the reference range (based on 2.5th–97.5th percentiles for this population) and users of potentially immuno-modulating medication, i.e., systemic corticosteroids, antiepileptic drugs, angiotensin converting enzyme inhibitors, cytostatics, immunomodulators, and/or immunosuppressants. Since *APOE* ε4 carrier status is a known risk factor of dementia with both central and systemic effects, we stratified by *APOE* ε4 carrier status.

After exclusion of participants with prevalent dementia, Cox proportional hazard regression analyses were performed to obtain hazard ratios (HRs) and 95% CIs for the association between serum Igs and incident dementia. Participants were followed from baseline to incident dementia, death, loss to follow-up, or January 1, 2016, whichever came first. Three models were constructed for the Cox analyses, which included the same covariables as the models for prevalent dementia with the addition of study cohort to adjust for temporal trends. The proportional hazards assumption was checked statistically and visually through the Schoenfeld test. A sensitivity analysis was performed after exclusion of participants with Ig levels outside the reference range and users of potentially immuno-modulating medication. We performed predefined stratification analyses by age (cut-off 65 years), sex, and *APOE* ε4 carrier status.

The association between serum Igs and cognitive test scores was assessed by linear regression analyses and presented as adjusted mean differences with 95% CIs. We included three models adjusting for similar potential confounders as in the analyses with prevalent dementia. Linearity was checked by ordinary least squares regression analyses with restricted cubic splines (three knots). In a sensitivity analysis, we excluded participants with Ig levels outside the reference range and users of potentially immuno-modulating medication. We furthermore stratified by sex.

The average percentage of missing values in covariables was 3.7% and these were imputed with multivariate imputation by chained equations (five imputations, ten iterations). Convergence was reached and the distribution of covariables before and after imputation was similar. All analyses were performed with R Statistical Software version 4.0.2. The PCA for G-factor was performed with IBM SPSS statistics version 24.0 (IBM Corp, Somers, NY).

## Results

We included 8,768 participants (median age 62.2 years; 57% women) with baseline serum Ig measurements and given informed consent for follow-up. Median levels of serum Igs were 2.10 g/L for IgA, 9.70 g/L for IgG, and 0.85 g/L for IgM. At baseline, 74 participants (0.8%) had dementia. Baseline characteristics are shown in Table 1.

**Table 1:** Baseline characteristics of 8,768 participants with serum immunoglobulin levels, information on dementia, and given informed consent for follow-up

***Serum immunoglobulins***^a^	
Serum IgA, g/L, median (IQR)	2.10 (1.57–2.78)
Serum IgG, g/L, median (IQR)	9.70 (8.30–11.20)
Serum IgM, g/L, median (IQR)	0.85 (0.59–1.23)
***Baseline cognition***^a,b^	
Prevalent dementia, *n* (%)	74 (0.8)
Global cognitive factor, *z*-score, median (IQR)	0.12 (-0.54–0.70)
Mini mental state examination, test score, median (IQR)	28 (27–29)
Letter digit substitution test, number of correct digits, median (IQR)	30 (25–35)
Stroop I test (inverted), seconds, median (IQR)	0.06 (0.05–0.07)
Stroop II test (inverted), seconds, median (IQR)	0.04 (0.04–0.05)
Stroop III test (inverted), seconds, median (IQR)	0.02 (0.02–0.03)
Word fluency test, number of animals, median (IQR)	22 (18–27)
Purdue pegboard left, number of pins placed, median (IQR)	13 (12–14)
Purdue pegboard right, number of pins placed, median (IQR)	14 (12–15)
Purdue pegboard both, number of pins placed, median (IQR)	11 (10–12)
Purdue pegboard sum, number of pins placed, median (IQR)	38 (35–41)
Word learning test (immediate), number of correct answers, median (IQR)	8.0 (6.7–9.7)
Word learning test (delayed), number of correct answers, median (IQR)	8.0 (6.0–10.0)
Word learning test (recognition), number of correct answers, median (IQR)	14.0 (13.0–15.0)
***Covariables***	
Sex, female, *n* (%)	4995 (57.0)
Age, years, median (IQR)	62.2 (57.4–70.7)
Smoking status, *n* (%)	
Never	2727 (31.1)
Former	4516 (51.5)
Current	1525 (17.4)
Alcohol consumption, *n* (%)	
None	1568 (17.9)
Mild (0–10 g/day)	4703 (53.6)
Moderate (10–20 g/day)	1424 (16.2)
Heavy (> 20 g/day)	1073 (12.2)
Highest education, *n* (%)	
Primary education	1044 (11.9)
Further education	6068 (69.2)
Higher education	1656 (18.9)
APOE ε4 carriers, *n* (%)	2496 (28.5)
BMI, kg/m^2^, median (IQR)	26.8 (24.5–29.7)
Hypertension, *n* (%)	3580 (40.8)
Diabetes mellitus, *n* (%)	1040 (11.9)
Stroke, *n* (%)	297 (3.4)
Coronary heart disease, *n* (%)	573 (6.5)
Serum cholesterol, mmol/L, mean (SD)	5.70 (1.03)
Users of immuno-modulating medication, *n* (%)	1385 (15.8)

Coronary heart disease comprises myocardial infarction, percutaneous coronary intervention, and/or coronary artery bypass grafting

Immunomodulating medication comprises systemic corticosteroids, antiepileptic drugs, angiotensin converting enzyme inhibitors, cytostatics, immunomodulators, and/or immunosuppressants;

*IgA* immunoglobulin A, *IgG* immunoglobulin G, *IgM* immunoglobulin M, *IQR* interquartile range, *BMI* body mass index, *SD* standard deviation

^a^Unstandardized values

^b^Available in a random subset of the study population

### Association between serum immunoglobulins and prevalent dementia

IgA was not associated with prevalent dementia, although a positive trend was seen (OR 1.12; 95% CI 0.92; 1.38). Within the reference range of IgA and after exclusion of users of immuno-modulating medication, effect estimates did not materially change (Table 2). For IgG, no association was found with prevalent dementia in the full range (i.e., levels across the entire measurable range) (OR 0.99; 95% CI 0.80; 1.21). After exclusion of participants with IgG levels outside the reference range and users of immuno-modulating medication, a negative trend was observed with prevalent dementia (OR 0.80; 95% CI 0.52; 1.24) (Table 2). IgM was not associated with prevalent dementia in either the full or reference range (Table 2).

**Table 2:** Association between standardized serum immunoglobulins and prevalent dementia

	*N* events/total	Odds Ratio (95% Confidence interval)		
		Model 1	Model 2	Model 3
IgA full range	74/8767	1.14 (0.96–1.36)	1.16 (0.97–1.40)	1.12 (0.92–1.38)
IgA reference range^a^	47/7030	1.07 (0.74–1.53)	1.11 (0.74–1.67)	1.07 (0.70–1.64)
IgG full range	74/8757	1.05 (0.88–1.25)	1.00 (0.83–1.21)	0.99 (0.80–1.21)
IgG reference range^a^	50/7029	0.82 (0.57–1.19)	0.80 (0.53–1.21)	0.80 (0.52–1.24)
IgM full range	74/8763	0.95 (0.76–1.20)	1.00 (0.79–1.25)	1.01 (0.81–1.27)
IgM reference range^a^	54/7062	1.05 (0.54–2.03)	1.10 (0.55–2.21)	1.07 (0.54–2.12)

Model 1 is adjusted for age and sex; model 2 is adjusted for model 1, smoking status, alcohol consumption, highest education, and *APOE* ε4 carrier status; model 3 is adjusted for model 2, body mass index, hypertension, total serum cholesterol, diabetes mellitus, history of coronary heart disease, and history of stroke

*IgA* immunoglobulin A, *IgG* immunoglobulin G, *IgM* immunoglobulin M

^a^Comprises 0.86–4.76 g/L for IgA, 6.20–15.10 g/L for IgG, and 0.28–2.64 g/L for IgM, and exclusion of users of medication known to influence serum immunoglobulin levels (systemic corticosteroids, antiepileptic drugs, angiotensin converting enzyme inhibitors, cytostatics, immuno-modulating and/or immunosuppressive drugs)

Higher IgA levels tended toward an increased odds of prevalent dementia in *APOE* ε4 carriers (OR 1.35; 95% CI 0.99; 1.84), although no statistically significant differences were observed between carriers and non-carriers. For IgG and IgM, no associations were seen in both *APOE* ε4 carriers and non-carriers (Supplementary Table S1).

### Association between serum immunoglobulins and incident dementia

Due to violation of the proportional hazards assumption, longitudinal associations were presented in follow-up strata in which the assumption was met (≤ 10 years and > 10 years).

Within the first 10 years of follow-up, no associations were seen between serum Igs and dementia risk. After exclusion of participants with Ig levels outside the reference range and users of immuno-modulating medication, effect estimates were generally attenuated except for IgM (Table 3).

**Table 3:** Association between standardized serum immunoglobulins and incident dementia

	*N* events/total	Hazard Ratio (95% Confidence interval)		
		Model 1	Model 2	Model 3
***Follow-up time ≤ 10 years***				
IgA full range	197/8,574	1.01 (0.89–1.14)	1.01 (0.89–1.14)	1.01 (0.89–1.14)
IgA reference range^a^	140/6,894	0.90 (0.72–1.11)	0.94 (0.76–1.16)	0.93 (0.75–1.15)
IgG full range	197/8,564	0.98 (0.87–1.10)	0.99 (0.88–1.12)	0.99 (0.87–1.11)
IgG reference range^a^	148/6,892	0.93 (0.76–1.14)	0.97 (0.79–1.18)	0.97 (0.79–1.19)
IgM full range	197/8,570	0.91 (0.78–1.06)	0.91 (0.78–1.06)	0.93 (0.80–1.08)
IgM reference range^a^	149/6,922	1.13 (0.79–1.61)	1.15 (0.81–1.64)	1.15 (0.81–1.64)
***Follow-up time > 10 years***				
IgA full range	509/4,436	1.00 (0.92–1.10)	1.01 (0.92–1.10)	1.00 (0.91–1.10)
IgA reference range^a^	415/3,666	1.03 (0.91–1.16)	1.04 (0.92–1.18)	1.04 (0.92–1.18)
IgG full range	509/4,429	0.99 (0.90–1.08)	0.99 (0.90–1.09)	0.99 (0.90–1.09)
IgG reference range^a^	421/3,641	0.95 (0.84–1.07)	0.94 (0.83–1.07)	0.94 (0.82–1.06)
IgM full range	509/4,433	0.97 (0.89–1.06)	0.97 (0.89–1.06)	0.97 (0.88–1.06)
IgM reference range^a^	414/3,661	0.81 (0.66–1.00)	0.81 (0.65–1.01)	**0.78 (0.63–0.97)**

Model 1 is adjusted for age, sex, and study cohort; model 2 is adjusted for model 1, smoking status, alcohol consumption, highest education, and *APOE* ε4 carrier status; model 3 is adjusted for model 2, body mass index, hypertension, total serum cholesterol, diabetes mellitus, history of coronary heart disease, and history of stroke. Follow-up time was divided into strata due to non-proportional hazards

Statistically significant associations (*P* value < 0.05) are in bold

*IgA* immunoglobulin A, *IgG* immunoglobulin G, *IgM* immunoglobulin M

^a^Comprises 0.86–4.76 g/L for IgA, 6.20–15.10 g/L for IgG, and 0.28–2.64 g/L for IgM, and exclusion of users of medication known to influence serum immunoglobulin levels (systemic corticosteroids, antiepileptic drugs, angiotensin converting enzyme inhibitors, cytostatics, immuno-modulating and/or immunosuppressive drugs)

After 10 years of follow-up, serum Igs were not associated with incident dementia overall. However, borderline significant associations were seen in certain subgroups. Within the reference range and after exclusion of users of immuno-modulating medication, higher IgM levels were associated with a decreased risk of dementia (HR 0.78; 95% CI 0.63; 0.97) (Table 3). Sex-stratified analyses mirrored the overall findings, with women driving the association between higher IgM levels and decreased dementia risk (Supplementary Table S2).

Stratification by age did not yield statistically significant differences between participants ≤ 65 and > 65 years for the association of serum Igs with dementia risk in either follow-up time stratum (Supplementary Table S2). When stratified by *APOE* ε4 carrier status, no associations were found for any Ig with dementia risk in either carriers or non-carriers regardless of follow-up duration (Supplementary Table S3).

### Association between serum immunoglobulins and cognition

**Fig. 1:**
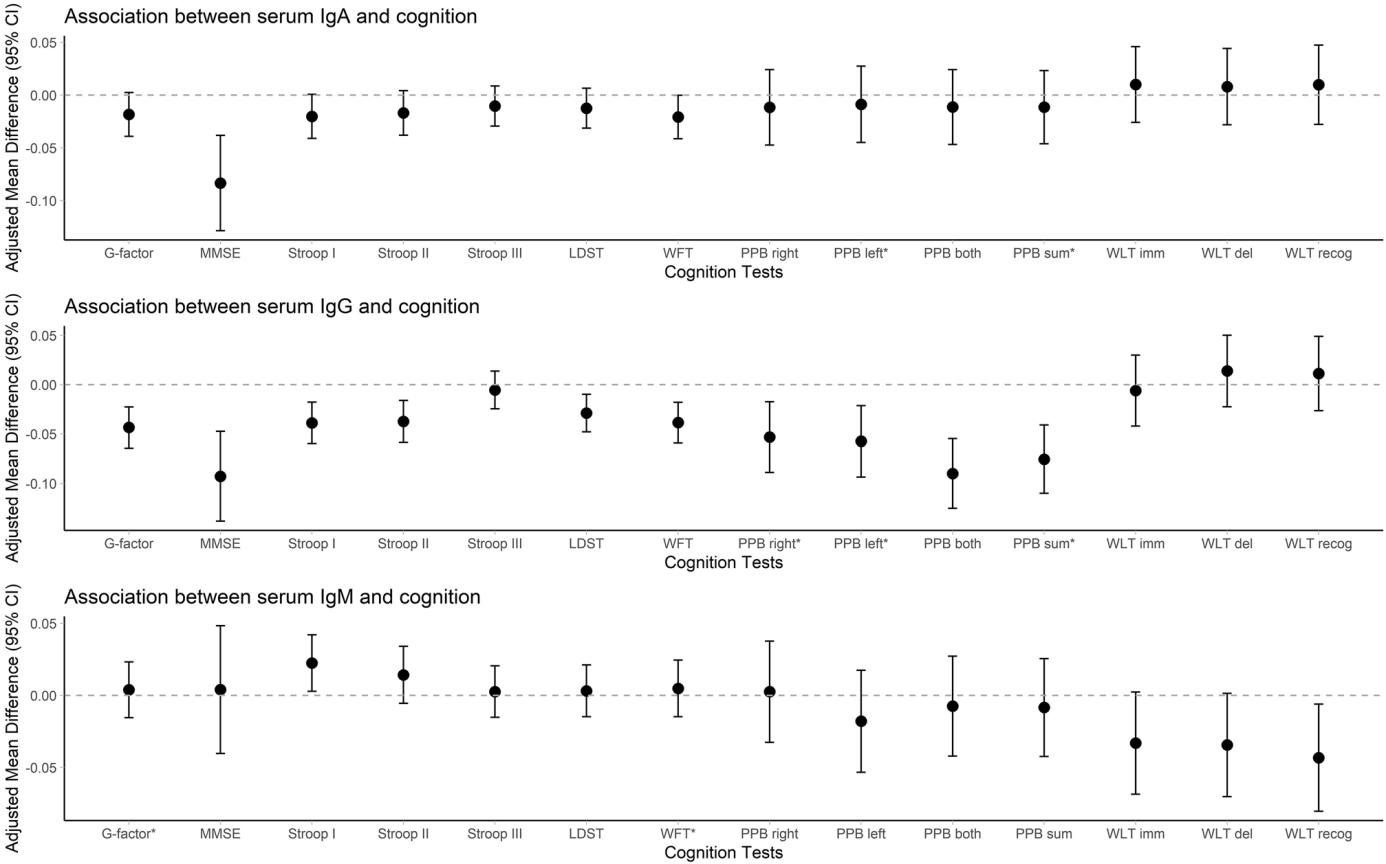
Associations between standardized serum immunoglobulins and cognition tests. Associations are adjusted for age, sex, smoking status, alcohol consumption, highest education, and *APOE* ε4 carrier status. Asterisks depict non-linear associations. Dots depict adjusted mean differences and vertical lines depict corresponding 95% confidence intervals. *IgA* immunoglobulin A, *IgG* immunoglobulin G, *IgM* immunoglobulin M, *95% CI* 95% confidence interval, *G-factor* global cognitive factor, *MMSE* mini mental state examination, *LDST* letter digit substitution test, *WFT* word fluency test, *PPB* Purdue pegboard test, *WLT* word learning test, *imm* immediate, *del* delayed, *recog* recognition

**Fig. 2:**
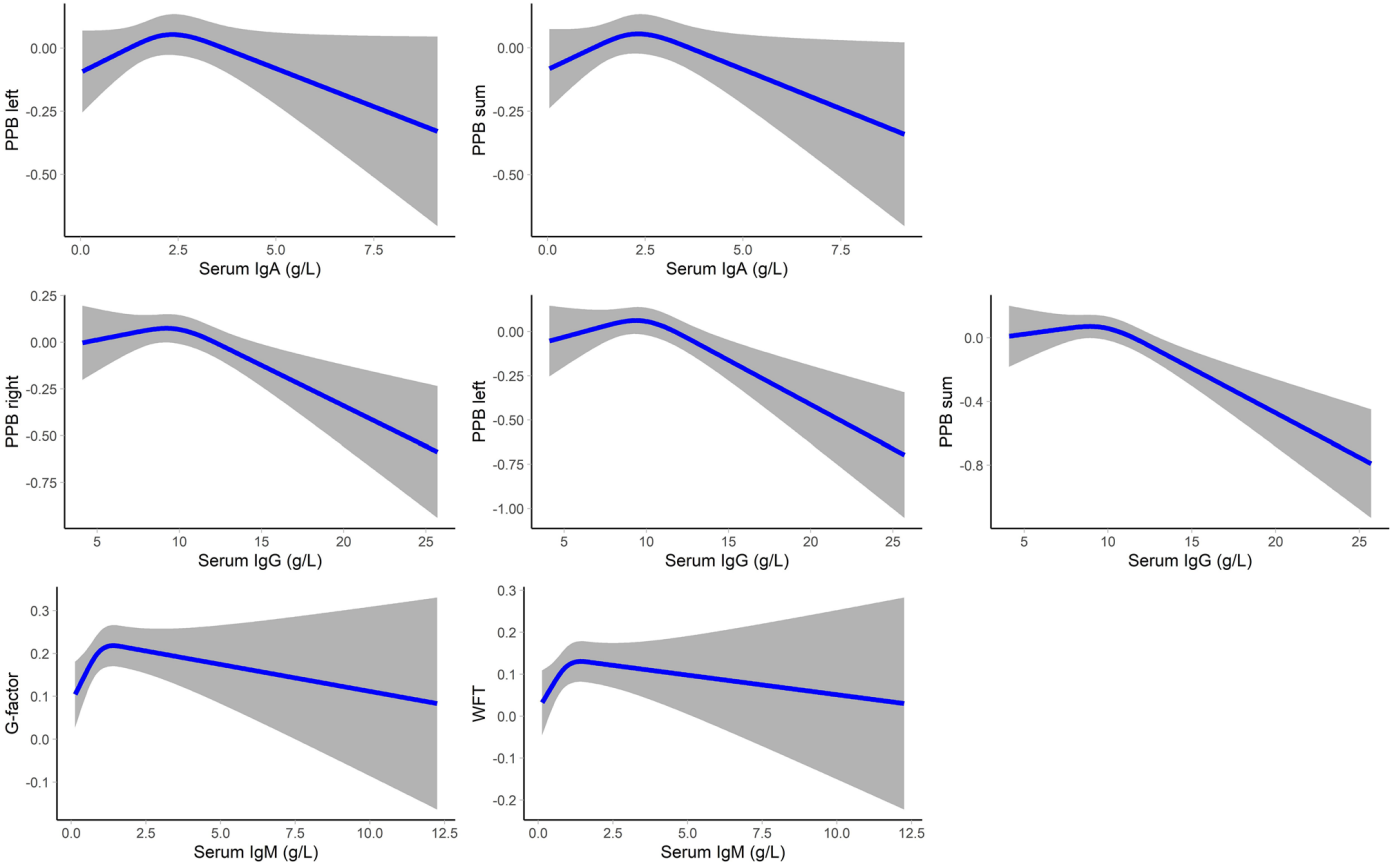
Non-linear associations between serum immunoglobulins and cognition tests. Associations are adjusted for age, sex, smoking status, alcohol consumption, highest education, and *APOE* ε4 carrier status. Lines depict adjusted mean differences and gray areas depict corresponding 95% confidence intervals. *IgA* immunoglobulin A, *IgG* immunoglobulin G, *IgM* immunoglobulin M, *PPB* Purdue pegboard test, *G-factor* global cognitive factor, *WFT* word fluency test

Higher IgA levels were associated with a lower MMSE score (adjusted mean difference − 0.075; 95% CI − 0.120; − 0.029). After exclusion of participants with IgA levels outside the reference range and users of immuno-modulating medication, higher IgA was associated with a lower WFT score (adjusted mean difference − 0.032; 95% CI − 0.061; − 0.002) and a higher WLT recognition score (adjusted mean difference 0.050; 95% CI 0.001; 0.099) (Supplementary Table S4). Higher IgG levels were associated with a lower score of most cognitive tests (adjusted mean differences between − 0.03 and − 0.09), although some associations were non-linear (Figs. 1, 2, Supplementary Table S4). Exclusion of participants with IgG levels outside the reference range and users of immuno-modulating medication yielded comparable results (Supplementary Table S4). For IgM, we only found associations within the full range. Higher IgM levels were associated with a higher Stroop I score (adjusted mean difference 0.021; 95% CI 0.002; 0.041) and a lower WLT recognition score (adjusted mean difference − 0.044; 95% CI − 0.081; − 0.007) (Fig. 1, Supplementary Table S4).

Stratification by sex did not reveal any clear differential cognitive patterns between men and women. Generally, higher IgG levels were associated with lower scores of most tests in both sexes. Results were heterogeneous for IgA and IgM (Supplementary Table S5).

## Discussion

In this large population-based cohort of middle-aged and older individuals, serum Ig levels were not associated with prevalent or incident dementia. Higher Ig levels (particularly serum IgG) were generally associated with worse cognitive test outcomes. However, due to the cross-sectional nature of the cognition test analyses, these results should be interpreted with caution.

Previously within the Rotterdam Study, general markers of systemic inflammation including granulocyte, lymphocyte, and platelet cell counts, were associated with an increased risk of dementia [16]. In the current study, we did not report associations of Igs with dementia risk. There are several possible explanations for a lack of association between serum Igs and dementia risk. First, it is possible that serum Igs have a negligible role in the pathophysiology of dementia, which would also be in line with the findings of previous RCTs. Second, it is possible that Igs are involved in local inflammatory processes (i.e., neuroinflammation), which may not be measurable in serum samples, but may be detectable in cerebrospinal fluid (CSF) samples instead. Due to an increased permeability of the blood–brain barrier in dementia, Igs may be elevated in the CSF due to filtration from the serum [18, 19]. A Swedish study reported higher IgG and IgM levels in the CSF of AD patients compared to healthy controls [10]. Aggregates of IgG have also been found in the corona of cerebral amyloid plaques [20, 21]. Third, it is possible that certain Ig subtypes or antigen-specific Igs are involved in the pathogenesis of dementia, whereas we only had information on total serum IgA, IgG, and IgM levels. A previous study on healthy aging reported higher anti-Aβ 42 (Aβ 42 being the most amyloidogenic peptide) IgM antibodies in healthy older blood donors (particularly women) compared to young controls. The authors furthermore found that anti-Aβ 42 IgM antibodies were lower in AD patients compared to age- and sex-matched controls, supporting the notion that these IgM antibodies contribute to heathy aging [22]. We did not find a consistent protective effect of IgM on dementia onset, suggesting that the measured Igs in our study cohort may have a low specificity to potential antigens involved in the pathophysiology of dementia. Although it has previously been argued that a lack of efficacy of IVIG in AD patients may be due to the inclusion of patients with an advanced disease stage [12], our results do not support this hypothesis, as there was no association between serum Igs and dementia risk in our population free from dementia at study baseline. This may imply that future trials should also consider other treatment options besides IVIG in dementia patients. Albumin is a large protein that can bind Aβ and inhibit Aβ fibrillization, but older age and AD are associated with structural and functional changes in albumin that render it less effective in the prevention of Aβ agglutination [23]. Recently, an RCT performed in patients with mild to moderate AD has shown that plasmapheresis with albumin replacement, with or without the addition of IVIG (to replace endogenous Ig removed by the plasmapheresis), is associated with less functional and cognitive decline [23].

Higher Ig levels were generally associated with a lower cognitive test score. This was most consistent for IgG. Previously, similar results have been reported, with higher IgA and IgG levels in cognitively impaired individuals compared to age- and sex-matched controls [9] and a negative association between the Weschler adult intelligence scale score and serum IgG in community-dwelling elderly [24]. Pro-inflammatory cytokines have been associated with worse memory, processing speed and motor function in elderly from the general population as well [25]. A review of studies performed in rodents showed that pro-inflammatory cytokines may impair cognition through a negative effect on synaptic plasticity and neurogenesis [26]. It is possible that elevated Ig levels reflect an ongoing inflammatory process with similar negative effects on the nervous system. However, our results on the association between serum Igs and cognitive function were cross-sectional. Therefore, it is unclear whether higher serum Ig levels lead to a worse cognition, or that they reflect pre-existing cognitive decline, since cognitive decline may result in a pro-inflammatory state through e.g., impairment of physical activity and dietary intake [27]. Furthermore, both elevated serum Igs and cognitive decline may be the result of a shared underlying cause. Future longitudinal studies are required to validate our findings.

If our results on the association between serum Igs and cognition indeed reflect reverse causation, one would expect a positive association between serum Igs and prevalent dementia as well. We did not demonstrate such an association. This could be due to a power issue as result of the relatively low number of prevalent dementia events in our cohort. Alternatively, pathophysiological differences between cognitive decline with retained independent functioning and dementia should be considered as well. In contrast to dementia, the blood–brain barrier permeability in mild cognitive impairment is unchanged [18]. This could imply a more important role of systemic rather than local inflammation in its pathophysiology. Serum Igs are not likely to cross into the central nervous system if the blood–brain barrier is still intact. In neurodegenerative mice with a disrupted blood–brain barrier, systemic inflammation was associated with an increased influx of serum IgG into the brain parenchyma. Furthermore, an increased cerebral expression of pro-inflammatory IgG-Fcγ receptors was reported [28]. Therefore, it is possible that clinical dementia is associated with neuro-inflammatory responses that cannot be measured in the serum.

Important strengths of our study were the large sample of community-dwelling middle-aged and older individuals, the long-term follow-up, and the extensive dementia ascertainment. We furthermore benefited from the availability of data on a wide range of potential confounders and performed additional analyses to take potential effect modification by age, sex, and *APOE* ε4 carrier status into account. Based on our findings, we cannot affirm pathophysiological grounds to apply IVIG treatment in individuals with pre-existing cognitive decline. Neither did we demonstrate that serum Igs may influence dementia onset over time. However, our study has certain limitations that should be addressed in future research. As mentioned above, we did not have information on Ig subclasses or antigen-specific Igs that may be involved in dementia. Furthermore, our study population is mainly Caucasian and therefore our results may not be generalizable to other populations. Lastly, studies with longitudinal data are necessary to prospectively assess the association between serum Igs and cognition in individuals from the general population.

## Supplementary Information

Below is the link to the electronic supplementary material.

Supplementary file1 (DOCX 33 KB)

## Data Availability

Data can be obtained upon request. Requests should be directed toward the management team of the Rotterdam Study (datamanagement.ergo@erasmusmc.nl), which has a protocol for approving data requests. Because of restrictions based on privacy regulations and informed consent of the participants, data cannot be made freely available in a public repository.
