# Supplementary material for: The association of serum immunoglobulins with cognition and dementia: the Rotterdam Study
Source: J Neurol. 2022 Sep 19;270(1):423–32. doi: 10.1007/s00415-022-11374-7 (PMC9813113; doi:10.1007/s00415-022-11374-7)
Supplement: Supplementary file 1 — Supplementary file1 (DOCX 33 KB) [file 415_2022_11374_MOESM1_ESM.docx]

**Supplementary Material “The association of serum immunoglobulins with cognition and dementia: the Rotterdam Study”**

Samer R. Khan MD^†^, Amber Yaqub MD^†^, M. Kamran Ikram MD PhD, P. Martin van Hagen MD PhD, Robin P. Peeters MD PhD, Virgil A.S.H. Dalm MD PhD, Layal Chaker MD PhD, M. Arfan Ikram MD PhD^*^

^†^These authors contributed equally to this work.

**^*^Corresponding author**

M. Arfan Ikram, MD PhD

Department of Epidemiology, Erasmus MC

PO Box 2040
3000 CA Rotterdam, the Netherlands

Tel: +31 10 70 43488 / 43391 / 43489

Fax: +31 10 70 44657

Email: [m.a.ikram@erasmusmc.nl](mailto:m.a.ikram@erasmusmc.nl)

| **Supplementary Table S1. Association between standardized serum immunoglobulins and prevalent dementia stratified by *APOE* ε4 carrier status** | | | | |
| --- | --- | --- | --- | --- |
|  | ***APOE* ε4 carriers** | | ***APOE* ε4 non-carriers** | |
|  | *N events/total* | *OR (95% CI)* | *N events/total* | *OR (95% CI)* |
| **IgA** | 30/2,495 | 1.35 (0.99-1.84) | 44/6,272 | 1.00 (0.74-1.36) |
| **IgG** | 30/2,495 | 0.94 (0.68-1.31) | 44/6,262 | 1.04 (0.79-1.38) |
| **IgM** | 30/2,495 | 0.85 (0.40-1.78) | 44/6,268 | 1.04 (0.80-1.36) |
| ORs are adjusted for age, sex, smoking status, alcohol consumption, highest education, BMI, hypertension, total serum cholesterol, diabetes mellitus, history of coronary heart disease, and history of stroke.  *P* value for interaction >.10 for all analyses.  OR, odds ratio; 95% CI, 95% confidence interval; IgA, immunoglobulin A; IgG, immunoglobulin G; IgM, immunoglobulin M; BMI, body mass index. | | | | |

| **Supplementary Table S2. Association between standardized serum immunoglobulins and incident dementia stratified by age and sex** | | | |
| --- | --- | --- | --- |
| **Follow-up time ≤10 years** | | | |
|  | **Hazard Ratio (95% Confidence Interval)**  **(N events/total)** | | |
|  | *IgA* | *IgG* | *IgM* |
| **Men** | 1.11 (0.92-1.36)  (69/3,702) | 0.90 (0.71-1.15)  (69/3,699) | 0.87 (0.61-1.23)  (69/3,700) |
| **Women** | 0.95 (0.81-1.12)  (128/4,872) | 1.03 (0.89-1.18)  (128/4,865) | 0.96 (0.80-1.15)  (128/4,870) |
| **Age ≤65 years** | **1.46 (1.03-2.06)**  (20/5,223) | 0.97 (0.57-1.63)  (20/5,216) | 0.81 (0.35-1.85)  (20/5,221) |
| **Age >65 years** | 1.06 (0.93-1.21)  (177/3,351) | 1.08 (0.95-1.22)  (177/3,348) | 0.91 (0.75-1.11)  (177/3,349) |
| **Follow-up time >10 years** | | | |
|  | **Hazard Ratio (95% Confidence Interval)**  **(N events/total)** | | |
|  | *IgA* | *IgG* | *IgM* |
| **Men** | 0.94 (0.81-1.10)  (176/1,854) | 1.10 (0.94-1.28)  (176/1,853) | 1.05 (1.00-1.11)^*^  (176/1,854) |
| **Women** | 1.03 (0.92-1.16)  (333/2,582) | 0.93 (0.83-1.05)  (333/2,576) | **0.71 (0.59-0.87)**^*^  (333/2,579) |
| **Age ≤65 years** | 0.89 (0.66-1.21)  (70/1,942) | 0.97 (0.73-1.30)  (70/1,938) | 0.93 (0.61-1.42)  (70/1,940) |
| **Age >65 years** | 1.03 (0.93-1.13)  (439/2,494) | 1.01 (0.91-1.12)  (439/2,491) | 0.96 (0.87-1.06)  (439/2,493) |
| ^*^*P* value for interaction <.10.  Effect estimates stratified by age are adjusted for sex, study cohort, smoking status, alcohol consumption, highest education, *APOE* ε4 carrier status, body mass index, hypertension, total serum cholesterol, diabetes mellitus, history of coronary heart disease, and history of stroke.  Effect estimates stratified by sex are adjusted for age, study cohort, smoking status, alcohol consumption, highest education, *APOE* ε4 carrier status, body mass index, hypertension, total serum cholesterol, diabetes mellitus, history of coronary heart disease, and history of stroke.  Statistically significant associations (*P* value <.05) are in bold.  IgA, immunoglobulin A; IgG, immunoglobulin G; IgM, immunoglobulin M. | | | |

| **Supplementary Table S3. Association between standardized serum immunoglobulins and incident dementia stratified by *APOE* ε4 carrier status** | | | | |
| --- | --- | --- | --- | --- |
| **Follow-up time ≤10 years** | | | | |
|  | ***APOE* ε4 carriers** | | ***APOE* ε4 non-carriers** | |
|  | *N events/total* | *HR (95% CI)* | *N events/total* | *HR (95% CI)* |
| **IgA** | 76/2,453 | 1.02 (0.83-1.26) | 121/6,121 | 0.99 (0.85-1.17) |
| **IgG** | 76/2,453 | 1.04 (0.84-1.29) | 121/6,111 | 0.97 (0.82-1.13) |
| **IgM** | 76/2,453 | 0.89 (0.63-1.25) | 121/6,117 | 0.93 (0.78-1.11) |
| **Follow-up time >10 years** | | | | |
|  | ***APOE* ε4 carriers** | | ***APOE* ε4 non-carriers** | |
|  | *N events/total* | *HR (95% CI)* | *N events/total* | *HR (95% CI)* |
| **IgA** | 219/1,212 | 0.97 (0.84-1.12) | 290/3,224 | 1.03 (0.91-1.16) |
| **IgG** | 219/1,212 | 0.90 (0.76-1.05) | 290/3,217 | 1.05 (0.93-1.18) |
| **IgM** | 219/1,212 | 0.82 (0.65-1.03)^*^ | 290/3,221 | 1.02 (0.94-1.10)^*^ |
| ^*^*P* value for interaction <.10.  HRs are adjusted for age, sex, study cohort, smoking status, alcohol consumption, highest education, BMI, hypertension, total serum cholesterol, diabetes mellitus, history of coronary heart disease, and history of stroke.  HR, hazard ratio; 95% CI, 95% confidence interval; IgA, immunoglobulin A; IgG, immunoglobulin G; IgM, immunoglobulin M; BMI, body mass index. | | | | |

| **Supplementary Table S4. Association between standardized serum immunoglobulins and cognition tests** | | | | | | |
| --- | --- | --- | --- | --- | --- | --- |
|  | **Adjusted mean difference (95% confidence interval)** | | | | | |
|  | *IgA full range* | *IgA reference range^a^* | *IgG full range* | *IgG reference range^a^* | *IgM full range* | *IgM reference range^a^* |
| **G-factor** | N = 7,687 | N = 6,128 | N = 7,679 | N = 6,134 | N = 7,683 | N = 6,154 |
| *Model 1* | -0.013 (-0.035 – 0.009) | -0.020 (-0.050 – 0.009) | **-0.044 (-0.065 – -0.022)** | **-0.044 (-0.073 – -0.015)** | 0.006 (-0.014 – 0.027)^b^ | **0.060 (0.012 – 0.108)** |
| *Model 2* | -0.018 (-0.039 – 0.002) | -0.027 (-0.056 – 0.001) | **-0.044 (-0.064 – -0.023)** | **-0.047 (-0.075 – -0.019)** | 0.004 (-0.015 – 0.023)^b^ | 0.038 (-0.007 – 0.084) |
| *Model 3* | -0.008 (-0.028 – 0.013) | -0.016 (-0.045 – 0.012) | **-0.036 (-0.057 – -0.016)** | **-0.040 (-0.068 – -0.012)** | 0.003 (-0.016 – 0.022)^b^ | 0.037 (-0.009 – 0.082) |
| **MMSE** | N = 8,699 | N = 6,857 | N = 8,689 | N = 6,867 | N = 8,695 | N = 6,894 |
| *Model 1* | **-0.089 (-0.135 – -0.042)** | **-0.087 (-0.151 – -0.024)** | **-0.109 (-0.155 – -0.063)** | **-0.072 (-0.134 – -0.009)** | 0.011 (-0.035 – 0.057) | 0.059 (-0.046 – 0.164) |
| *Model 2* | **-0.083 (-0.129 – -0.038)** | **-0.085 (-0.147 – -0.024)** | **-0.093 (-0.138 – -0.047)** | -0.061 (-0.123 – 0.002) | 0.004 (-0.040 – 0.048) | 0.032 (-0.071 – 0.135) |
| *Model 3* | **-0.075 (-0.120 – -0.029)** | **-0.082 (-0.144 – -0.020)** | **-0.089 (-0.135 – -0.044)** | -0.062 (-0.125 – 0.001) | 0.003 (-0.041 – 0.047) | 0.031 (-0.072 – 0.134) |
| **Stroop I** | N = 7,895 | N = 6,262 | N = 7,887 | N = 6,272 | N = 7,891 | N = 6,294 |
| *Model 1* | -0.017 (-0.038 – 0.005) | -0.017 (-0.046 – 0.012) | **-0.038 (-0.059 – -0.017)** | -0.027 (-0.056 – 0.002) | **0.025 (0.005 – 0.045)** | 0.031 (-0.017 – 0.079) |
| *Model 2* | -0.020 (-0.041 – 0.001) | -0.021 (-0.050 – 0.008) | **-0.039 (-0.060 – -0.018)** | **-0.030 (-0.059 – -0.001)** | **0.022 (0.003 – 0.042)** | 0.015 (-0.032 – 0.062) |
| *Model 3* | -0.013 (-0.034 – 0.008) | -0.013 (-0.042 – 0.016) | **-0.035 (-0.056 – -0.014)** | -0.027 (-0.056 – 0.002) | **0.021 (0.002 – 0.041)** | 0.014 (-0.033 – 0.061) |
| **Stroop II** | N = 7,885 | N = 6,256 | N = 7,877 | N = 6,266 | N = 7,881 | N = 6,289 |
| *Model 1* | -0.013 (-0.034 – 0.008) | -0.012 (-0.042 – 0.018) | **-0.034 (-0.055 – -0.012)** | -0.019 (-0.049 – 0.010) | 0.016 (-0.004 – 0.036) | 0.004 (-0.045 – 0.052) |
| *Model 2* | -0.017 (-0.038 – 0.004) | -0.017 (-0.047 – 0.012) | **-0.037 (-0.059 – -0.016)** | -0.026 (-0.056 – 0.003) | 0.014 (-0.006 – 0.034) | -0.007 (-0.055 – 0.041) |
| *Model 3* | -0.010 (-0.031 – 0.011) | -0.010 (-0.039 – 0.020) | **-0.034 (-0.055 – -0.012)** | -0.022 (-0.052 – 0.007) | 0.013 (-0.007 – 0.033) | -0.009 (-0.057 – 0.039) |
| **Stroop III** | N = 7,864 | N = 6,244 | N = 7,856 | N = 6,253 | N = 7,860 | N = 6,276 |
| *Model 1* | -0.007 (-0.026 – 0.013) | -0.011 (-0.038 – 0.016) | -0.002 (-0.021 – 0.018) | 0.015 (-0.012 – 0.042) | 0.005 (-0.013 – 0.024) | 0.001 (-0.043 – 0.045) |
| *Model 2* | -0.010 (-0.029 – 0.009) | -0.018 (-0.044 – 0.009) | -0.006 (-0.025 – 0.014) | 0.006 (-0.020 – 0.033) | 0.003 (-0.015 – 0.020) | -0.015 (-0.058 – 0.028) |
| *Model 3* | -0.004 (-0.023 – 0.015) | -0.009 (-0.036 – 0.017) | -0.003 (-0.022 – 0.017) | 0.010 (-0.017 – 0.036) | 0.002 (-0.016 – 0.019) | -0.016 (-0.059 – 0.027) |
| **LDST** | N = 7,935 | N = 6,290 | N = 7,927 | N = 6,300 | N = 7,931 | N = 6,321 |
| *Model 1* | -0.008 (-0.028 – 0.012) | 0.000 (-0.027 – 0.028) | **-0.026 (-0.046 – -0.006)** | -0.026 (-0.053 – 0.001) | 0.006 (-0.012 – 0.025) | 0.023 (-0.022 – 0.068) |
| *Model 2* | -0.012 (-0.031 – 0.007) | -0.005 (-0.032 – 0.022) | **-0.029 (-0.048 – -0.010)** | **-0.033 (-0.059 – -0.006)** | 0.003 (-0.015 – 0.021) | 0.004 (-0.039 – 0.047) |
| *Model 3* | -0.007 (-0.026 – 0.012) | 0.001 (-0.026 – 0.027) | **-0.027 (-0.046 – -0.008)** | **-0.032 (-0.058 – -0.005)** | 0.003 (-0.015 – 0.020) | 0.003 (-0.039 – 0.046) |
| **WFT** | N = 8,009 | N = 6,335 | N = 8,001 | N = 6,345 | N = 8,005 | N = 6,366 |
| *Model 1* | -0.019 (-0.040 – 0.002) | **-0.033 (-0.063 – -0.004)** | **-0.040 (-0.061 – -0.019)** | **-0.044 (-0.073 – -0.014)** | 0.007 (-0.013 – 0.027)^b^ | **0.050 (0.002 – 0.098)** |
| *Model 2* | -0.021 (-0.041 – -0.000) | **-0.037 (-0.066 – -0.008)** | **-0.039 (-0.059 – -0.018)** | **-0.044 (-0.073 – -0.015)** | 0.005 (-0.015 – 0.025)^b^ | 0.034 (-0.013 – 0.081) |
| *Model 3* | -0.016 (-0.037 – 0.004) | **-0.032 (-0.061 – -0.002)** | **-0.036 (-0.056 – -0.015)** | **-0.042 (-0.071 – -0.012)** | 0.004 (-0.015 – 0.024)^b^ | 0.033 (-0.014 – 0.080) |
| **PPB right** | N = 2,991 | N = 2,381 | N = 2,988 | N = 2,415 | N = 2,991 | N = 2,403 |
| *Model 1* | -0.002 (-0.038 – 0.034) | -0.009 (-0.055 – 0.038) | -0.031 (-0.067 – 0.005)^b^ | -0.000 (-0.047 – 0.046)^b^ | 0.004 (-0.031 – 0.040) | 0.005 (-0.073 – 0.083) |
| *Model 2* | -0.012 (-0.047 – 0.024) | -0.025 (-0.071 – 0.022) | **-0.053 (-0.089 – -0.018)^b^** | -0.029 (-0.077 – 0.019) | 0.003 (-0.033 – 0.038) | -0.009 (-0.087 – 0.069) |
| *Model 3* | -0.004 (-0.040 – 0.031) | -0.016 (-0.063 – 0.031) | **-0.049 (-0.084 – -0.013)^b^** | -0.019 (-0.067 – 0.028) | 0.000 (-0.035 – 0.035) | -0.011 (-0.088 – 0.067) |
| **PPB left** | N = 2,986 | N = 2,382 | N = 2,983 | N = 2,415 | N = 2,986 | N = 2,402 |
| *Model 1* | 0.003 (-0.033 – 0.040)^b^ | 0.002 (-0.045 – 0.049)^b^ | -0.036 (-0.071 – 0.000)^b^ | **-0.029 (-0.076 – 0.018)^b^** | -0.017 (-0.053 – 0.018) | -0.017 (-0.096 – 0.061) |
| *Model 2* | -0.009 (-0.045 – 0.027)^b^ | -0.015 (-0.061 – 0.032) | **-0.058 (-0.094 – -0.021)^b^** | **-0.061 (-0.108 – -0.013)^b^** | -0.018 (-0.053 – 0.017) | -0.031 (-0.109 – 0.047) |
| *Model 3* | -0.002 (-0.038 – 0.034)^b^ | -0.006 (-0.052 – 0.041) | **-0.053 (-0.089 – -0.017)^b^** | **-0.050 (-0.098 – -0.003)^b^** | -0.020 (-0.055 – 0.015) | -0.034 (-0.111 – 0.044) |
| **PPB both** | N = 2,977 | N = 2,375 | N = 2,974 | N = 2,408 | N = 2,977 | N = 2,396 |
| *Model 1* | -0.003 (-0.039 – 0.032)^b^ | -0.004 (-0.051 – 0.042)^b^ | **-0.072 (-0.107 – -0.037)^b^** | **-0.052 (-0.098 – -0.006)** | -0.007 (-0.042 – 0.028) | 0.024 (-0.054 – 0.101) |
| *Model 2* | -0.011 (-0.047 – 0.024) | -0.018 (-0.064 – 0.028)^b^ | **-0.090 (-0.125 – -0.055)** | **-0.077 (-0.124 – -0.031)** | -0.007 (-0.042 – 0.027) | 0.010 (-0.067 – 0.087) |
| *Model 3* | -0.005 (-0.040 – 0.030)^b^ | -0.008 (-0.054 – 0.038)^b^ | **-0.086 (-0.121 – -0.051)** | **-0.068 (-0.115 – -0.021)** | -0.009 (-0.044 – 0.025) | 0.008 (-0.069 – 0.085) |
| **PPB sum** | N = 2,971 | N = 2,371 | N = 2,968 | N = 2,404 | N = 2,971 | N = 2,392 |
| *Model 1* | 0.000 (-0.035 – 0.035)^b^ | -0.004 (-0.049 – 0.041)^b^ | **-0.052 (-0.086 – -0.017)^b^** | **-0.029 (-0.074 – 0.017)^b^** | -0.008 (-0.042 – 0.027) | 0.003 (-0.072 – 0.079) |
| *Model 2* | -0.011 (-0.046 – 0.023)^b^ | -0.021 (-0.067 – 0.023) | **-0.076 (-0.110 – -0.041)^b^** | **-0.061 (-0.107 – -0.016)^b^** | -0.008 (-0.042 – 0.026) | -0.012 (-0.087 – 0.063) |
| *Model 3* | -0.004 (-0.038 – 0.030)^b^ | -0.011 (-0.056 – 0.034)^b^ | **-0.070 (-0.104 – -0.036)^b^** | **-0.050 (-0.095 – -0.004)^b^** | -0.011 (-0.044 – 0.023) | -0.014 (-0.089 – 0.060) |
| **WLT imm** | N = 3,041 | N = 2,412 | N = 3,038 | N = 2,446 | N = 3,041 | N = 2,436 |
| *Model 1* | 0.013 (-0.024 – 0.049) | 0.026 (-0.023 – 0.075) | -0.003 (-0.039 – 0.033) | 0.017 (-0.032 – 0.066) | -0.029 (-0.065 – 0.008) | -0.003 (-0.084 – 0.078) |
| *Model 2* | 0.010 (-0.026 – 0.046) | 0.019 (-0.029 – 0.067) | -0.006 (-0.042 – 0.030) | 0.016 (-0.033 – 0.064) | -0.033 (-0.069 – 0.002) | -0.034 (-0.113 – 0.045) |
| *Model 3* | 0.014 (-0.022 – 0.050) | 0.027 (-0.021 – 0.074) | -0.004 (-0.040 – 0.032) | 0.024 (-0.024 – 0.073) | -0.034 (-0.070 – 0.001) | -0.036 (-0.115 – 0.043) |
| **WLT del** | N = 3,040 | N = 2,411 | N = 3,037 | N = 2,445 | N = 3,040 | N = 2,435 |
| *Model 1* | 0.007 (-0.030 – 0.044) | 0.022 (-0.026 – 0.071) | 0.013 (-0.023 – 0.050) | 0.027 (-0.022 – 0.076) | -0.030 (-0.067 – 0.006) | -0.008 (-0.089 – 0.074) |
| *Model 2* | 0.008 (-0.028 – 0.044) | 0.020 (-0.028 – 0.068) | 0.014 (-0.023 – 0.050) | 0.028 (-0.021 – 0.078) | -0.034 (-0.070 – 0.001) | -0.033 (-0.113 – 0.047) |
| *Model 3* | 0.011 (-0.025 – 0.047) | 0.027 (-0.021 – 0.075) | 0.015 (-0.021 – 0.052) | 0.036 (-0.013 – 0.086) | -0.036 (-0.072 – -0.000) | -0.035 (-0.115 – 0.045) |
| **WLT recog** | N = 3,041 | N = 2,412 | N = 3,038 | N = 2,446 | N = 3,041 | N = 2,436 |
| *Model 1* | 0.010 (-0.028 – 0.048) | **0.053 (0.004 – 0.102)** | 0.012 (-0.026 – 0.049) | 0.010 (-0.040 – 0.060) | **-0.040 (-0.078 – -0.003)** | -0.006 (-0.088 – 0.076) |
| *Model 2* | 0.010 (-0.028 – 0.047) | **0.050 (0.001 – 0.099)** | 0.011 (-0.027 – 0.049) | 0.008 (-0.042 – 0.059) | **-0.043 (-0.080 – -0.006)** | -0.023 (-0.105 – 0.059) |
| *Model 3* | 0.010 (-0.028 – 0.047) | **0.050 (0.001 – 0.099)** | 0.010 (-0.028 – 0.048) | 0.008 (-0.042 – 0.059) | **-0.044 (-0.081 – -0.007)** | -0.023 (-0.105 – 0.059) |
| ^a^Comprises 0.86-4.76 g/L for IgA, 6.20-15.10 g/L for IgG, and 0.28-2.64 g/L for IgM, and exclusion of participants using medication known to influence serum immunoglobulin levels (systemic corticosteroids, antiepileptic drugs, angiotensin converting enzyme inhibitors, cytostatics, immunomodulating and/or immunosuppressive drugs).  ^b^Non-linear associations.  Model 1 is adjusted for age and sex; model 2 is adjusted for model 1, smoking status, alcohol consumption, highest education, and *APOE* ε4 carrier status; model 3 is adjusted for model 2, body mass index, hypertension, total serum cholesterol, diabetes mellitus, history of coronary heart disease, and history of stroke.  All cognition tests with the exception of G-factor and MMSE were standardized for analyses. The Stroop tests were furthermore inverted for interpretation.  Statistically significant associations (*P* value <.05) are in bold.  IgA, immunoglobulin A; IgG, immunoglobulin G; IgM, immunoglobulin M; MMSE, mini mental state examination; LDST, letter digit substitution test; WFT, word fluency test; PPB, purdue pegboard test; WLT, word learning test; imm, immediate; del, delayed; recog, recognition. | | | | | | |

| **Supplementary Table S5. Association between standardized serum immunoglobulins and cognition tests stratified by sex** | | | | | | |
| --- | --- | --- | --- | --- | --- | --- |
|  | **Adjusted mean difference (95% confidence interval)** | | | | | |
|  | *IgA Men* | *IgA Women* | *IgG Men* | *IgG Women* | *IgM Men* | *IgM Women* |
| **G-factor** | N = 3,287 | N = 4,400 | N = 3,284 | N = 4,395 | N = 3,285 | N = 4,398 |
| *Model 1* | -0.003 (-0.034 – 0.028) | -0.023 (-0.053 – 0.007) | **-0.045 (-0.079 – -0.011)** | **-0.046 (-0.074 – -0.018)** | 0.001 (-0.023 – 0.025) | 0.006 (-0.030 – 0.041)^a^ |
| *Model 2* | -0.012 (-0.042 – 0.018) | -0.024 (-0.053 – 0.005) | **-0.053 (-0.086 – -0.020)** | **-0.041 (-0.068 – -0.013)** | 0.002 (-0.022 – 0.025) | -0.001 (-0.035 – 0.033)^a^ |
| *Model 3* | -0.006 (-0.035 – 0.024) | -0.009 (-0.038 – 0.020) | **-0.049 (-0.082 – -0.017)** | **-0.032 (-0.060 – -0.005)** | 0.001 (-0.022 – 0.025) | -0.002 (-0.036 – 0.031)^a^ |
| **MMSE** | N = 3,746 | N = 4,953 | N = 3,743 | N = 4,946 | N = 3,744 | N = 4,951 |
| *Model 1* | 0.014 (-0.046 – 0.074) | **-0.180 (-0.249 – -0.112)** | **-0.070 (-0.135 – -0.004)** | **-0.136 (-0.199 – -0.073)** | -0.003 (-0.053 – 0.047) | 0.024 (-0.060 – 0.108) |
| *Model 2* | 0.008 (-0.050 – 0.066) | **-0.164 (-0.231 – -0.096)** | **-0.068 (-0.133 – -0.004)** | **-0.112 (-0.175 – -0.049)** | -0.006 (-0.055 – 0.043) | 0.011 (-0.070 – 0.093) |
| *Model 3* | 0.011 (-0.047 – 0.069) | **-0.147 (-0.215 – -0.079)** | **-0.066 (-0.131 – -0.001)** | **-0.105 (-0.168 – -0.042)** | -0.008 (-0.056 – 0.040) | 0.010 (-0.072 – 0.091) |
| **Stroop I** | N = 3,395 | N = 4,500 | N = 3,392 | N = 4,495 | N = 3,393 | N = 4,498 |
| *Model 1* | -0.008 (-0.038 – 0.023) | -0.024 (-0.054 – 0.005)^a^ | -0.029 (-0.063 – 0.004) | **-0.044 (-0.071 – -0.017)** | 0.012 (-0.012 – 0.036) | **0.051 (0.016 – 0.086)** |
| *Model 2* | -0.014 (-0.044 – 0.016) | -0.025 (-0.054 – 0.004)^a^ | -0.032 (-0.065 – 0.001) | **-0.044 (-0.071 – -0.016)** | 0.013 (-0.011 – 0.036) | **0.046 (0.012 – 0.081)** |
| *Model 3* | -0.011 (-0.041 – 0.019) | -0.013 (-0.042 – 0.016)^a^ | -0.032 (-0.065 – 0.001) | **-0.037 (-0.065 – -0.010)** | 0.011 (-0.013 – 0.035) | **0.045 (0.011 – 0.080)** |
| **Stroop II** | N = 3,386 | N = 4,499 | N = 3,383 | N = 4,494 | N = 3,384 | N = 4,497 |
| *Model 1* | -0.007 (-0.037 – 0.023) | -0.018 (-0.048 – 0.012) | -0.033 (-0.066 – -0.000) | **-0.033 (-0.061 – -0.005)** | 0.013 (-0.011 – 0.037) | 0.026 (-0.009 – 0.062) |
| *Model 2* | -0.012 (-0.041 – 0.018) | -0.021 (-0.051 – 0.008) | **-0.035 (-0.068 – -0.002)** | **-0.038 (-0.066 – -0.010)** | 0.013 (-0.011 – 0.037) | 0.022 (-0.013 – 0.057) |
| *Model 3* | -0.008 (-0.038 – 0.021) | -0.009 (-0.039 – 0.020) | -0.033 (-0.066 – -0.000) | **-0.033 (-0.061 – -0.005)** | 0.012 (-0.012 – 0.035) | 0.021 (-0.014 – 0.056) |
| **Stroop III** | N = 3,377 | N = 4,487 | N = 3,374 | N = 4,482 | N = 3,375 | N = 4,485 |
| *Model 1* | 0.012 (-0.016 – 0.040) | -0.023 (-0.050 – 0.004) | 0.014 (-0.016 – 0.044) | -0.012 (-0.037 – 0.013) | -0.003 (-0.025 – 0.019) | 0.022 (-0.010 – 0.054) |
| *Model 2* | 0.006 (-0.021 – 0.033) | -0.024 (-0.051 – 0.002) | 0.005 (-0.025 – 0.035) | -0.011 (-0.036 – 0.014) | -0.003 (-0.025 – 0.018) | 0.016 (-0.015 – 0.047) |
| *Model 3* | 0.009 (-0.018 – 0.036) | -0.013 (-0.040 – 0.013) | 0.005 (-0.025 – 0.035) | -0.007 (-0.032 – 0.019) | -0.004 (-0.026 – 0.017) | 0.015 (-0.016 – 0.046) |
| **LDST** | N = 3,415 | N = 4,520 | N = 3,412 | N = 4,515 | N = 3,413 | N = 4,518 |
| *Model 1* | -0.005 (-0.033 – 0.022) | -0.011 (-0.039 – 0.016) | -0.025 (-0.055 – 0.005) | **-0.029 (-0.055 – -0.003)** | -0.001 (-0.023 – 0.021) | 0.012 (-0.022 – 0.045) |
| *Model 2* | -0.014 (-0.040 – 0.013) | -0.011 (-0.038 – 0.016) | **-0.036 (-0.065 – -0.007)** | **-0.027 (-0.052 – -0.002)** | -0.002 (-0.023 – 0.019) | 0.005 (-0.027 – 0.037) |
| *Model 3* | -0.011 (-0.037 – 0.016) | -0.003 (-0.030 – 0.024) | **-0.035 (-0.064 – -0.006)** | -0.024 (-0.050 – 0.001) | -0.003 (-0.024 – 0.018) | 0.005 (-0.027 – 0.037) |
| **WFT** | N = 3,448 | N = 4,561 | N = 3,445 | N = 4,556 | N = 3,446 | N = 4,559 |
| *Model 1* | -0.011 (-0.042 – 0.019) | -0.027 (-0.056 – 0.002) | -0.026 (-0.060 – 0.007) | **-0.050 (-0.076 – -0.023)** | 0.010 (-0.015 – 0.035) | -0.001 (-0.036 – 0.034)^a^ |
| *Model 2* | -0.016 (-0.046 – 0.014)^a^ | -0.023 (-0.051 – 0.006) | **-0.034 (-0.067 – -0.001)** | **-0.040 (-0.067 – -0.014)** | 0.009 (-0.015 – 0.034) | -0.008 (-0.042 – 0.025)^a^ |
| *Model 3* | -0.015 (-0.045 – 0.015)^a^ | -0.014 (-0.042 – 0.015) | -0.034 (-0.067 – -0.000) | **-0.036 (-0.062 – -0.009)** | 0.009 (-0.015 – 0.034) | -0.009 (-0.043 – 0.025)^a^ |
| **PPB right** | N = 1,285 | N = 1,706 | N = 1,283 | N = 1,705 | N = 1,284 | N = 1,707 |
| *Model 1* | -0.009 (-0.064 – 0.047) | 0.004 (-0.044 – 0.051) | -0.043 (-0.101 – 0.015) | -0.023 (-0.067 – 0.022)^a^ | 0.000 (-0.047 – 0.047) | 0.009 (-0.046 – 0.064) |
| *Model 2* | -0.018 (-0.073 – 0.037) | -0.006 (-0.054 – 0.041) | **-0.067 (-0.125 – -0.009)** | -0.043 (-0.089 – 0.002)^a^ | -0.001 (-0.047 – 0.046**)** | 0.006 (-0.049 – 0.061) |
| *Model 3* | -0.015 (-0.070 – 0.040) | 0.005 (-0.042 – 0.052) | **-0.066 (-0.124 – -0.009)** | -0.036 (-0.082 – 0.009)^a^ | -0.005 (-0.051 – 0.041) | 0.005 (-0.049 – 0.060) |
| **PPB left** | N = 1,284 | N = 1,702 | N = 1,282 | N = 1,701 | N = 1,283 | N = 1,703 |
| *Model 1* | -0.004 (-0.060 – 0.052)^a^ | 0.007 (-0.040 – 0.055) | **-0.061 (-0.120 – -0.002)^a^** | -0.020 (-0.065 – 0.025)^a^ | 0.014 (-0.034 – 0.061) | **-0.066 (-0.121 – -0.011)** |
| *Model 2* | -0.016 (-0.071 – 0.040)^a^ | -0.003 (-0.051 – 0.045) | **-0.089 (-0.147 – -0.030)** | -0.034 (-0.080 – 0.011)^a^ | 0.014 (-0.033 – 0.061) | **-0.067 (-0.122 – -0.013)** |
| *Model 3* | -0.014 (-0.069 – 0.041)^a^ | 0.007 (-0.040 – 0.055) | **-0.089 (-0.148 – -0.031)** | -0.026 (-0.071 – 0.019)^a^ | 0.010 (-0.037 – 0.057) | **-0.068 (-0.122 – -0.014)** |
| **PPB both** | N = 1,278 | N = 1,699 | N = 1,276 | N = 1,698 | N = 1,277 | N = 1,700 |
| *Model 1* | -0.018 (-0.074 – 0.038)^a^ | 0.007 (-0.040 – 0.053) | **-0.087 (-0.145 – -0.028)^a^** | **-0.064 (-0.107 – -0.020)** | -0.002 (-0.049 – 0.046) | -0.017 (-0.071 – 0.037) |
| *Model 2* | -0.029 (-0.084 – 0.026)^a^ | 0.001 (-0.046 – 0.047) | **-0.109 (-0.167 – -0.050)** | **-0.078 (-0.122 – -0.034)** | -0.001 (-0.047 – 0.046) | -0.017 (-0.070 – 0.036) |
| *Model 3* | -0.027 (-0.081 – 0.028)^a^ | 0.011 (-0.035 – 0.058) | **-0.107 (-0.165 – -0.049)** | **-0.069 (-0.113 – -0.025)** | -0.005 (-0.051 – 0.042) | -0.017 (-0.069 – 0.036) |
| **PPB sum** | N = 1,276 | N = 1,695 | N = 1,274 | N = 1,694 | N = 1,275 | N = 1,696 |
| *Model 1* | -0.012 (-0.067 – 0.043)^a^ | 0.008 (-0.038 – 0.054) | **-0.073 (-0.130 – -0.016)** | -0.039 (-0.082 – 0.004)^a^ | 0.004 (-0.042 – 0.051) | -0.028 (-0.081 – 0.024) |
| *Model 2* | -0.024 (-0.078 – 0.029)^a^ | -0.002 (-0.048 – 0.043) | **-0.101 (-0.157 – -0.044)** | **-0.058 (-0.101 – -0.014)^a^** | 0.005 (-0.040 – 0.051) | -0.030 (-0.082 – 0.022) |
| *Model 3* | -0.022 (-0.075 – 0.032)^a^ | 0.009 (-0.036 – 0.054) | **-0.100 (-0.156 – -0.044)** | **-0.049 (-0.092 – -0.006)^a^** | 0.001 (-0.045 – 0.046) | -0.030 (-0.082 – 0.021) |
| **WLT imm** | N = 1,310 | N = 1,731 | N = 1,308 | N = 1,730 | N = 1,309 | N = 1,732 |
| *Model 1* | **0.068 (0.014 – 0.121)** | -0.029 (-0.079 – 0.022) | 0.005 (-0.052 – 0.061) | -0.007 (-0.054 – 0.041) | 0.006 (-0.041 – 0.052) | **-0.074 (-0.133 – -0.016)** |
| *Model 2* | **0.067 (0.015 – 0.119)** | -0.030 (-0.079 – 0.020) | -0.003 (-0.059 – 0.053) | -0.004 (-0.051 – 0.044) | -0.001 (-0.046 – 0.045) | **-0.075 (-0.132 – -0.019)** |
| *Model 3* | **0.069 (0.017 – 0.121)** | -0.025 (-0.074 – 0.025) | -0.004 (-0.060 – 0.052) | -0.002 (-0.049 – 0.046) | -0.002 (-0.048 – 0.043) | **-0.076 (-0.132 – -0.019)** |
| **WLT del** | N = 1,310 | N = 1,730 | N = 1,308 | N = 1,729 | N = 1,309 | N = 1,731 |
| *Model 1* | 0.045 (-0.008 – 0.099) | -0.021 (-0.071 – 0.030) | 0.008 (-0.048 – 0.065) | 0.018 (-0.029 – 0.066) | 0.000 (-0.046 – 0.047) | **-0.069 (-0.127 – -0.011)^a^** |
| *Model 2* | 0.047 (-0.006 – 0.099) | -0.018 (-0.068 – 0.032) | 0.005 (-0.051 – 0.062) | 0.022 (-0.025 – 0.070) | -0.005 (-0.051 – 0.041) | **-0.070 (-0.127 – -0.013)^a^** |
| *Model 3* | 0.048 (-0.005 – 0.101) | -0.013 (-0.063 – 0.037) | 0.003 (-0.053 – 0.060) | 0.024 (-0.024 – 0.072) | -0.006 (-0.052 – 0.039) | **-0.071 (-0.128 – -0.014)** |
| **WLT recog** | N = 1,310 | N = 1,731 | N = 1,308 | N = 1,730 | N = 1,309 | N = 1,732 |
| *Model 1* | **0.078 (0.017 – 0.138)** | -0.041 (-0.089 – 0.007) | 0.014 (-0.050 – 0.078) | 0.012 (-0.033 – 0.057) | -0.012 (-0.065 – 0.041) | **-0.077 (-0.132 – -0.021)^a^** |
| *Model 2* | **0.077 (0.016 – 0.137)** | -0.042 (-0.089 – 0.006) | 0.013 (-0.052 – 0.078) | 0.014 (-0.032 – 0.059) | -0.014 (-0.067 – 0.038) | **-0.079 (-0.134 – -0.024)** |
| *Model 3* | **0.078 (0.018 – 0.139)** | -0.044 (-0.092 – 0.004) | 0.013 (-0.051 – 0.078) | 0.010 (-0.036 – 0.056) | -0.015 (-0.067 – 0.038) | **-0.080 (-0.135 – -0.026)** |
| ^a^Non-linear associations.  Model 1 is adjusted for age; model 2 is adjusted for model 1, smoking status, alcohol consumption, highest education, and *APOE* ε4 carrier status; model 3 is adjusted for model 2, body mass index, hypertension, total serum cholesterol, diabetes mellitus, history of coronary heart disease, and history of stroke.  All cognition tests with the exception of G-factor and MMSE were standardized for analyses. The Stroop tests were furthermore inverted for interpretation.  Statistically significant associations (*P* value <.05) are in bold.  IgA, immunoglobulin A; IgG, immunoglobulin G; IgM, immunoglobulin M; MMSE, mini mental state examination; LDST, letter digit substitution test; WFT, word fluency test; PPB, purdue pegboard test; WLT, word learning test; imm, immediate; del, delayed; recog, recognition. | | | | | | |
